# Sugar Industry Influence on the Scientific Agenda of the National Institute of Dental Research’s 1971 National Caries Program: A Historical Analysis of Internal Documents

**DOI:** 10.1371/journal.pmed.1001798

**Published:** 2015-03-10

**Authors:** Cristin E. Kearns, Stanton A. Glantz, Laura A. Schmidt

**Affiliations:** 1 Philip R. Lee Institute for Health Policy Studies, University of California San Francisco, San Francisco, California, United States of America; 2 Department of Medicine, University of California San Francisco, San Francisco, California, United States of America; 3 Department of Orofacial Sciences, University of California San Francisco, San Francisco, California, United States of America; 4 Center for Tobacco Control Research and Education, University of California San Francisco, San Francisco, California, United States of America; 5 Helen Diller Family Comprehensive Cancer Center, University of California San Francisco, San Francisco, California, United States of America; 6 Clinical and Translational Science Institute, University of California San Francisco, San Francisco, California, United States of America; 7 Department of Anthropology, History and Social Medicine, University of California San Francisco, San Francisco, California, United States of America; University of Liverpool, UNITED KINGDOM

## Abstract

**Background:**

In 1966, the National Institute of Dental Research (NIDR) began planning a targeted research program to identify interventions for widespread application to eradicate dental caries (tooth decay) within a decade. In 1971, the NIDR launched the National Caries Program (NCP). The objective of this paper is to explore the sugar industry’s interaction with the NIDR to alter the research priorities of the NIDR NCP.

**Methods and Findings:**

We used internal cane and beet sugar industry documents from 1959 to 1971 to analyze industry actions related to setting research priorities for the NCP. The sugar industry could not deny the role of sucrose in dental caries given the scientific evidence. They therefore adopted a strategy to deflect attention to public health interventions that would reduce the harms of sugar consumption rather than restricting intake. Industry tactics included the following: funding research in collaboration with allied food industries on enzymes to break up dental plaque and a vaccine against tooth decay with questionable potential for widespread application, cultivation of relationships with the NIDR leadership, consulting of members on an NIDR expert panel, and submission of a report to the NIDR that became the foundation of the first request for proposals issued for the NCP. Seventy-eight percent of the sugar industry submission was incorporated into the NIDR’s call for research applications. Research that could have been harmful to sugar industry interests was omitted from priorities identified at the launch of the NCP. Limitations are that this analysis relies on one source of sugar industry documents and that we could not interview key actors.

**Conclusions:**

The NCP was a missed opportunity to develop a scientific understanding of how to restrict sugar consumption to prevent tooth decay. A key factor was the alignment of research agendas between the NIDR and the sugar industry. This historical example illustrates how industry protects itself from potentially damaging research, which can inform policy makers today. Industry opposition to current policy proposals—including a World Health Organization guideline on sugars proposed in 2014 and changes to the nutrition facts panel on packaged food in the US proposed in 2014 by the US Food and Drug Administration—should be carefully scrutinized to ensure that industry interests do not supersede public health goals.

## Abstract 2

In a historical analysis of internal documents, Stanton Glantz and colleagues examine the sugar industry influence of the National Institute of Dental Research's 1971 National Caries Program.

## Editors' Summary

**Background.:**

Tooth decay (dental caries) is the leading chronic disease of children and adolescents. Although largely preventable, 42% of children in the US have some decay in their baby (primary) teeth, and 59% of adolescents have cavities in their permanent teeth. Tooth decay occurs when the hard enamel covering the tooth surface is damaged by acid, which is produced by bacteria in the mouth. Plaque, a sticky substance of bacteria, food particles, and saliva, constantly forms on teeth. When you eat food—particularly sugary foods and drinks—the bacteria in plaque produce acids that attack the tooth enamel. The stickiness of the plaque keeps the acids in contact with the teeth. Plaque buildup can be prevented by regular brushing and flossing. Dentists can detect tooth decay before it causes toothache through visual examination or by taking dental X-rays, and can treat the condition by removing the decay and plugging the hole with a “dental filling.” However, if the decay has damaged the nerve in the center of the tooth, root canal treatment or removal of the tooth may be necessary.

**Why Was This Study Done?:**

Experts generally agree that sugars play a causal role in tooth decay. Consequently, in 2014, the World Health Organization (WHO) issued a draft guideline that recommended a daily limit on the consumption of “free” sugars (sugars added to food by manufacturers, cooks, or consumers). Also in 2014, the US Food and Drug Administration (FDA) proposed that the nutrition facts panels on US packaged food products should list added sugars. As with similar proposals made in the past, the World Sugar Research Organisation, a trade organization that represents companies with economic interests in sugar production, is challenging these proposals, arguing that, rather than trying to limit sugar intake, public health interventions to prevent tooth decay should focus on reducing the harms of sugar consumption. Here, the researchers explore how the sugar industry has historically sought to undermine or subvert policies to restrict sugar consumption, by examining internal industry documents related to the launch of a targeted research program to identify interventions to eradicate tooth decay—the National Caries Program (NCP)—by the US National Institute of Dental Research (NIDR) in 1971.

**What Did the Researchers Do and Find?:**

The researchers analyzed an archive of 319 internal sugar industry documents from 1959 to 1971 (the “Roger Adams papers”) and NIDR documents to explore how the sugar industry sought to influence the setting of research priorities for the NCP. Their analysis indicates that, as early as 1950, sugar industry trade organizations had accepted that sugar damaged teeth and had recognized that the dental community favored restricting sugar intake as a key way to control caries. The sugar industry therefore adopted a strategy to deflect attention towards public health interventions that would reduce the harms of sugar consumption. This strategy included tactics such as funding research into enzymes that break up dental plaque and into a vaccine against tooth decay, and cultivating relationships with the NIDR leadership. Notably, 78% of a report submitted to the NIDR by the sugar industry was directly incorporated into the NIDR’s first request for research proposals for the NCP, and research that could have been harmful to sugar industry interests (specifically, research into methods to measure the propensity of specific foods to cause caries) was omitted from the research priorities identified at the launch of the NCP.

**What Do These Findings Mean?:**

These findings, although limited by the researchers’ reliance on a single source of industry documents and by the absence of interviews with key actors in the launch of the NCP, reveal an alignment of research agendas between the NIDR and the sugar industry in the early 1970s. The findings also suggest that the NCP was a missed opportunity to develop a scientific understanding of how to restrict sugar consumption to prevent tooth decay. Indeed, although tooth decay declined by 20% between 1971/1973 and 1980, 64% of children still developed caries a decade after the NCP was launched. Most importantly, these findings illustrate how the sugar industry has protected itself from potentially damaging research in the past; a similar approach has also been taken by the tobacco industry. These findings highlight the need to carefully scrutinize industry opposition to the proposed WHO and FDA guidelines on sugar intake and labeling, respectively, to ensure that industry interests do not interfere with current efforts to improve dental public health.

**Additional Information.:**

Please access these websites via the online version of this summary at http://dx.doi.org/10.1371/journal.pmed.1001798.

- The US National Institute of Dental and Craniofacial Research (the successor to the NIDR) provides detailed information on tooth decay (in English and Spanish)
- The US Centers for Disease Control and Prevention also provides information on dental caries
- The UK National Health Service Choices website provides detailed information about all aspects of tooth decay; it also provides an analysis of a recent news report concerning research supporting the proposed WHO guideline for limiting sugar intake
- MedlinePlus provides links to additional information about tooth decay (in English and Spanish)
- Information about the 2014 WHO draft guideline on sugar intake and about the changes proposed to the nutrition facts label by the FDA are available (in English and Spanish)

## Introduction

Despite overwhelming consensus on the causal role of sugars in tooth decay [1] and recommendations by expert committees [2–4], quantitative targets restricting the intake of sugars to control dental caries have not been widely implemented [5]. In 2003, a joint committee of the World Health Organization (WHO) and the Food and Agriculture Organization (FAO) recommended limiting “free” or added sugars, defined as “monosaccharides and disaccharides added to foods by the manufacturer, cook or consumer, and sugars naturally present in honey, syrups, fruit juices and fruit concentrates” to 10% of total calories [3]. The World Sugar Research Organisation (WSRO), a trade organization representing more than 30 international members with economic interests in the cane and beet sugar industry, including the Sugar Association (SA) in the US and Coca-Cola [6], successfully blocked the 2003 WHO/FAO joint committee recommendation from becoming WHO policy [7]. The WHO/FAO joint committee quantitative recommendation to limit free sugars [3] was replaced with the nonspecific recommendation to “limit the intake of free sugars” [8]. In 2014, based largely on the global burden of dental disease, the WHO Nutrition Guidance Expert Advisory Group issued draft guidelines with strong quantitative recommendations to limit daily consumption of free sugars to 10% of total calories, with a further suggestion to limit free sugars to less than 5% of total calories [4]. As with the 2003 WHO recommendation, WSRO and its members have submitted comments in opposition to the 2014 WHO draft recommendation [9,10] and have signaled willingness to contest the 2014 recommendations with equal force as in 2003 [11,12]. WSRO argued that dental public health interventions should focus on reducing the harm of sugar consumption with methods such as the “regular use of fluoride toothpaste” rather than restricting sugar intake [9,13].

Publications about food industry influence on public health policy are growing [14–21], but analyses of food industry documents are rare [22]. Historical analyses of internal tobacco industry documents have proven key to informing policy and litigation successes in tobacco control [23–27]. There are similar historical internal documents related to WSRO that could inform public health efforts by illuminating sugar industry activities designed to undermine or subvert policies to restrict sugar consumption [28].

We analyzed previously unexplored sugar industry documents to trace industry interactions with the US National Institute of Dental Research (NIDR, which changed its name to the National Institute of Dental and Craniofacial Research [NIDCR] in 1998) between 1966 and 1971, a critical period for dental caries control policy when the NIDR planned the launch of the National Caries Program (NCP) with the goal of eradicating dental caries within one decade [29]. Reflecting the research priorities of the sugar industry, the 1971 NCP research priorities ignored strategies to limit sugar consumption and focused instead on fluoride delivery, reducing the virulence of oral bacteria, and modifying food products with additives to counter sugar’s harmful effects [30]. Ultimately, the NCP, which drove the US dental caries research agenda for more than a decade, failed to significantly reduce the burden of dental caries [31], a preventable disease that remains the leading chronic disease in children and adolescents in the US [32].

## Methods

### Data Sources

#### Sugar industry documents

**Fig 1:**
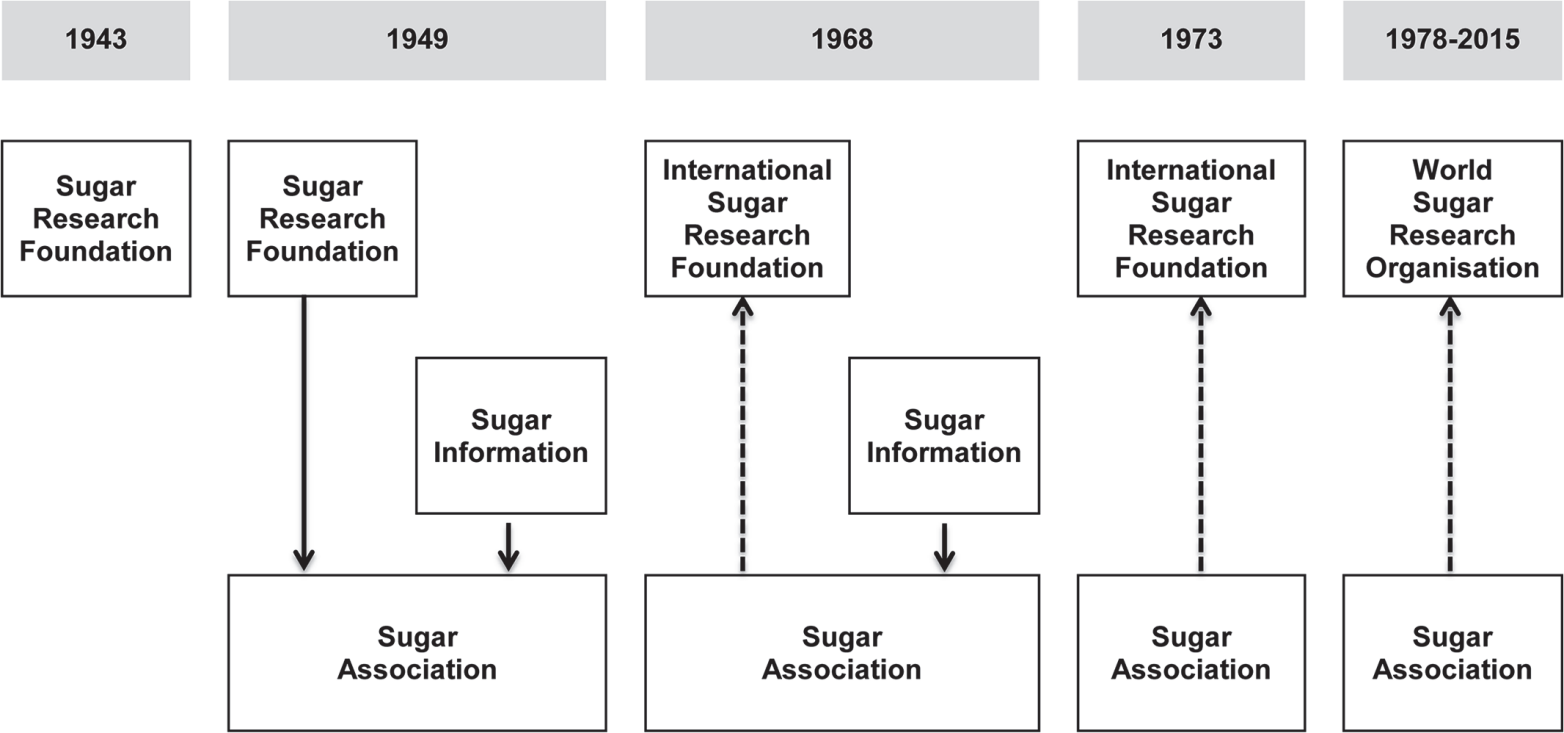
Two sugar industry organizations operating as of 2015, the World Sugar Research Organisation and the Sugar Association, evolved out of the Sugar Research Foundation. In 1943, SRF was founded in New York, New York. In 1949, SA was created to oversee the research activities of SRF (the research arm) and the newly created Sugar Information (the public relations arm). In 1968, SRF dissociated from SA and was reorganized as ISRF. SA joined ISRF as a member (shown as a dotted line). In 1973, SA discontinued Sugar Information because there was no longer a meaningful separation of duties between SA and Sugar Information. In 1978, ISRF was reorganized to become WSRO, and SA joined WSRO as a member.

This study drew substantially on previously unexplored WSRO-related internal documents from between 1959 and 1971 [33]. WSRO was formed from a number of related sugar industry trade organizations including the Sugar Research Foundation (SRF) and the International Sugar Research Foundation (ISRF) (Fig. 1) [6,34–36]. The first author located these documents in 2010 in an inventory of the papers of Roger Adams housed in the University of Illinois Archives through a Google search using the terms “International Sugar Research Foundation” and “archives” [33]. Roger Adams, Emeritus Professor of Organic Chemistry, served on the SRF and then ISRF Scientific Advisory Board [37] from 1959 until his death in 1971 [38,39]. Adams’s files contain correspondence with sugar industry executives, meeting minutes, and other relevant reports. After reviewing the inventory of the Roger Adams papers and consulting with University of Illinois archivists, the first author identified 319 documents (1,551 pages) related to SRF/ISRF. Additional material authored by SRF, ISRF, and WSRO was located through a WorldCat search, including annual reports, symposium proceedings, and reviews of research. Documents were carefully reviewed for relevance to dental caries research and policy.

#### National Institute of Dental Research documents

We located sources related to the NIDR NCP through searches of PubMed and WorldCat, and by contacting NIDCR directly. Materials included NCP primary publications [40–45] and two historical reviews commissioned by the NIDR: a description of the first decade of the NCP by its project officer, William E. Rogers [29], and a history of the NIDR by historian Ruth Roy Harris [31].

Findings were assembled chronologically into a narrative case study. Part of the analysis called for systematically comparing two key reports for similarities: (1) *Dental Caries Research—1969* [46], a document submitted by ISRF to the NIDR, and (2) the NIDR’s 1971 *Opportunities for Participation in the National Caries Program* [30], which defined the research priorities at the launch of the NCP. Both documents were entered into Microsoft Word using a monospaced font at 12 characters per inch (average of 12 words per line). After line numbering both documents, we compared the documents, classifying each line of the 1971 NIDR document and the 1969 ISRF document as different, paraphrased, or verbatim. “Paraphrased” was defined as some identical words with the same overall meaning.

## Results

### Emergence of the National Caries Program, 1966–1967

Table 1 provides a timeline of events during the planning and launch of the NCP.

**Table 1:** Timeline of events of sugar industry influence on the scientific agenda of the National Institute of Dental Research’s 1971 National Caries Program.

Key Dates	NIDR	SRF and ISRF
1959		Roger Adams becomes member of SRF Scientific Advisory Board [37]
June 1966	NIDR Director Seymour Kreshover initiates planning for what would become NCP [29,31]	
1967		SRF funds Project 269 to develop dextranase enzyme and vaccine [47]
June 1968	Announcement of Caries Task Force [31]	Philip Ross (with ties to the US National Institutes of Health) elected ISRF president [48,49], coordinates meetings with the NIDR prior to NCP launch [50]
June 1969		Symposium on the Status of Research in Sucrochemistry, Diet and Heart Disease, Obesity, Dental Caries, and Clinical Nutrition held; Prof. G. Neil Jenkins speaks on “Sugar and Dental Caries” [51]
Sept. 1969		Symposium held: Seeking New Approaches to Old Problems; the NIDR’s Richard Greulich speaks on “The Future of Caries Control” [52]
Oct. 1969	Caries Task Force Steering Committee meeting on research priorities; planning for Role of Human Foodstuffs in Caries Workshop Conference [29]	ISRF convenes Panel Meeting of the Dental Caries Task Force—members of the NIDR Caries Task Force Steering Committee participate [53]
Late 1969		Submission of ISRF report *Dental Caries Research—1969* to the NIDR Caries Task Force [46]
Jan. 1970	NIDR Laboratory of Microbiology chief Henry Scherp submits *A National Caries Program of the National Institute of Dental Research*: *Ten-Year Program of Research and Development*; Nixon selects NCP as special health initiative to be funded in fiscal year 1971 [41]	
Feb. 1970	President Nixon endorses NCP [31]	Celebratory *International Sugar Research Foundation Special Report*: *Dental Caries* mailed to Roger Adams [50]
March 1970	Caries Task Force holds Role of Human Foodstuffs in Caries Workshop Conference [42]	
March 1971	NCP becomes operational [29]; Omnibus request for contracts, *Opportunities for Participation in the National Caries Program*, released [30]	

In June 1966, President Lyndon Johnson initiated a major reappraisal of National Institutes of Health (NIH) research agendas, requesting that directors of NIH institutes submit their programs’ “priorities and objectives in the national attack on disease and disability” [29]. The NIDR Director Seymour Kreshover’s report to President Johnson in November 1966 stated that “an accelerated program of research during the next decade could reasonably provide the means for virtual eradication of dental caries” [31].

The threat of the NIDR’s dental research program to the sugar industry began to crystallize in July 1967, after the president praised Kreshover’s report [31]. While it had long been known that bacteria caused tooth decay [54], Kreshover based his plans on the work of NIDR scientists Robert Fitzgerald and Paul Keyes, who had singled out the bacterial strain *Streptococcus mutans* as a major culprit in the production of acids that caused dental caries [55,56]. Research suggested that sucrose was more hazardous than other types of sugars because it caused *S*. *mutans* to form dextrans, sticky molecules that caused the bacteria to tenaciously adhere to one another in the plaque and on the tooth’s surface [57]. The NIDR’s increased interest in *S*. *mutans* brought renewed scrutiny to sucrose consumption and dental caries risk.

In October 1967, the NIDR’s National Dental Advisory Council identified three main areas of emphasis to inform research priorities to eradicate caries: reducing the virulence of bacteria once exposed to sugars, fluoride delivery, and, of most concern to the sugar industry, dietary modification [31]. A particular threat was research conducted by NIDR scientist Robert Stephan, initiated in the 1940s, on the “cariogenic” (decay-causing) potential of foods [58–60]. According to Stephan, as of 1966:

There have been a great many observations, discussions, and controversies published in the literature concerning the role of different foods and particularly sweets in the etiology [of dental caries]. However…there seems to be little controlled experimental proof to show which foods are cariogenic and which noncariogenic in humans. [61]

Stephan had initiated work to develop an animal model that could “evaluate cariogenicity and anticariogenicity of different foods and beverages that people like and commonly consume” [61]. Based on existing research at the time, foods containing sucrose were in danger of being placed at the top of the list of harmful cariogenic products [62].

### Industry Deflection of Attention Away from Limiting Sugar Intake

#### Industry position on caries control

At least as early as 1950, SRF knew its product damaged teeth and appreciated that both the scientific evidence and the dental community favored restricting sugar intake as a key way to control caries [63]. The 1950 SRF annual report stated:

The ultimate aim of the Foundation in dental research has been to discover effective means of controlling tooth decay *by methods other than restricting carbohydrate intake*. This program has both laboratory and clinical aspects.

*There is evidence tending to show that carbohydrates*, *including sugar*, *and perhaps other food types*, *are implicated in tooth decay*. There is also evidence, though less convincing, that soluble sugars may play a bigger role than starches. Besides the relatively clear evidence there are many conjectures, traditions and myths that confuse the picture.

*Until recently the great majority of the dental profession had adopted the view that practical control of tooth decay could be achieved only by restriction of carbohydrates*, *particularly sugar in the diet*. Scientific logic, nevertheless, points to many other promising possibilities and many of these are supported by preliminary laboratory observations. [63] (emphasis added)

The 1950 SRF annual report also shows that industry research was selected as part of a strategy to deflect attention away from sugar restriction as a means to control caries [63].

#### Funding research to divert attention from limiting sugar intake

Consistent with a deflection strategy, between 1967 and 1970, SRF funded Project 269 to bolster research on interventions not requiring sugar restriction to control dental caries [47]. Project 269, led by Professor Bertram Cohen at the Royal College of Surgeons of England, sought to render *S*. *mutans* less destructive to teeth after sugar was consumed using enzymes called dextranases to break the sticky dextrans in dental plaque formed after sugar was consumed [47]. Project 269 also attempted to develop a vaccine against tooth decay that would allow people to continue to consume sugar [47]. The NIDR had investigated both methods in the 1960s [31] and found that although dextranases added to the food and water of rodents had shown some promise of being effective, more research was necessary before human applications could be developed [64], and a vaccine against *S*. *mutans* tested in hamsters failed to prevent tooth decay [65]. By 1962, NIDR scientists were suggesting that measures other than a vaccine would be needed to control dental caries [31].

SRF allocated US$12,000 (US$85,455 in 2014 dollars) to Project 269 between 1967 and 1970 [47]. Project 269 was primarily funded by the chocolate and confectionary industries and had an annual budget of US$120,000 (US$854,558 in 2014 dollars) [47]. A confidential report mailed to Roger Adams summarizing Project 269 indicated that SRF considered dental caries “one of the major troublesome factors in the nonacceptance of sucrose” [47]. SRF leaders hoped that their support for this new project would prove a “significant way of solving the problem” [47].

Funding from SRF and the chocolate and confectionary industry allowed Cohen to create a new laboratory to use monkeys for the development of dextranases and a tooth decay vaccine for human application [47]. SRF hoped that the work on dextranases and a vaccine could be handed over to drug companies to develop commercial quantities [47]. A 1968 *Montreal Gazette* article, “These Monkeys May Save Your Teeth,” reported that one practical application for dextranase under consideration was “to mix it with raw sugar and use it as a powder on desserts and cakes and in soft drinks” [66]. Cohen was described as having “little sympathy for those who would ban sweet things,” and was quoted as saying “Why should people be denied pleasure? It would obviously be far better to eliminate the harmful effects” [66]. While at the time there was less attention paid to scientific conflicts of interest than in 2015, the article mentioned that a grant from the Nuffield Foundation funded the building of the research unit that housed the monkeys, but not that the sugar or chocolate and confectionary industries were also supporting Cohen’s work [66].

### Setting Research Priorities for the National Caries Program, 1968–1969

At a June 1968 press conference, NIDR Director Kreshover announced the creation of the Caries Task Force chaired by NIDR Laboratory of Microbiology chief Henry Scherp to develop the NCP [31]. A subcommittee, the Caries Task Force Steering Committee, was assigned the essential task of identifying research priorities [29]. Task force members were largely drawn from federal agencies and academia (Table 2). Professor Basil Bibby, with a strong background in developing models that could evaluate the cariogenicity of foods, would be assigned a leading role in evaluating research supporting dietary interventions to eliminate tooth decay [29].

**Table 2:** Comparison of membership of the NIDR Caries Task Force Steering Committee and ISRF Panel Meeting of Dental Caries Task Force.

Name	Affiliation	NIDR Caries Task Force Steering Committee, 1969 [31]	ISRF Panel Meeting of Dental Caries Task Force, October 20, 1969 [53]
Basil G. Bibby	Director, Eastman Dental Center	X	X
George W. Burnett	Professor of Microbiology, School of Dentistry, Medical College of Georgia	X	X
James P. Carlos	Chief, Biometry Section, NIDR		X
Charles J. Donnelly	Chief, Dental Caries and Hard Tissues Program, Extramural Programs, NIDR	X	X
Robert J. Fitzgerald	Laboratory of Microbiology, NIDR	X	
John C. Greene	Deputy Director, Division of Dental Health, Bureau of Health Professions, Education of Manpower Training, NIH	X	X
Robert S. Harris	Professor of Nutritional Biochemistry, Massachusetts Institute of Technology	X	X
John Knutson	Professor of Preventive Dentistry, School of Dentistry, University of California, Los Angeles	X	X
Bo Krasse	Professor of Cariology and Dean, Faculty of Odontology, University of Gothenburg, Sweden		X
Seymour Kreshover	Director, NIDR and Caries Task Force Steering Committee	X	X
Henry W. Scherp	Chief, Laboratory of Microbiology, NIDR, Chairman Caries Task Force	X	X

In 1968, SRF reorganized as ISRF to carry on SRF’s research mission at the global level [48]. Existing SRF research projects, including Project 269, continued to be supported by ISRF [67]. ISRF was also interested in engaging federal research agencies. On July 1, 1968, Dr. Philip Ross became ISRF president [48]. Ross had ties to the NIH, having served as chief of the NIDR/NIH Research Grants Section from 1963 to 1965, then as assistant head of the NIH Special International Programs Section until 1967 [49]. Moreover, that summer, ISRF moved its headquarters from New York to Bethesda, Maryland, near the NIH [68].

#### Industry reviews dental caries literature

As the NIDR Caries Task Force Steering Committee began meeting to discuss research priorities in 1969, ISRF scheduled a series of meetings to select “the areas of research that [ISRF] should be attacking” [69]. Table 3 provides an overview of the research priorities discussed by the NIDR and ISRF committees at key moments leading up to the launch of the NCP. According to ISRF President Ross, ISRF meetings would consider “critical reviews of the major areas [concerning] sugar,” including a range of public health topics: “dental caries, overweight and obesity, [and] atherosclerotic vascular disease” [69]. Panels of outside consultants would be convened, and the results of these activities compiled and sent to ISRF Scientific Advisory Board members by December 1969 [70].

**Table 3:** Comparison of Research Priorities Identified by ISRF and the NIDR, 1969–1971.

Feasible Interventions to Eradicate Dental Caries	(A) Prof. G. Neil Jenkins address to ISRF, “Sugar and Dental Caries,” June 1969 [51]	(B) NIDR’s Richard Greulich address to ISRF, “The Future of Caries Control,” September 1969 [52]	(C) NIDR Caries Task Force Steering Committee, October 1969 [29]	(D) ISRF Panel Meeting of the Dental Caries Task Force, October 1969 [71]	(E) ISRF Submission to the NIDR: *Dental Caries Research—1969*, Late 1969 [46]	(F) NIDR Caries Task Force Role of Human Foodstuffs in Caries Workshop Conference, March 1970 [72]	(G) NIDR Request for Contracts, *Opportunities for Participation in the National Caries Program*, 1971 [30]
**Dietary interventions**							
Cariogenic potential of foods			Deferred to March 1970 meeting			X	
Dietary phosphates	X	X	X	X	X	X	X
Invert sugars		X	X			X	X
Dietary trace elements	X		X	X		X	X
**Non-dietary interventions**							
Dextranase	X	X	X	X	X	N/A	X
Low molecular weight dextrans		X	X		X	N/A	X
Antimicrobial agents			X	X	X	N/A	X
Antibiotics			X		X	N/A	X
Immunization	X		X	X	X	N/A	X
Water fluoridation	X		X	X	X	N/A	
Topical application of fluoride	X		X		X	N/A	X
Addition of fluoride to sugar, salt, flour			X	X	X	N/A	
Sealants		X	X	X	X	N/A	X
**Other**						N/A	
Dental epidemiology			X			N/A	
Education for motivation			X			N/A	

N/A, not applicable.

ISRF launched its critical review of dental caries by inviting Dr. G. Neil Jenkins, a professor at the University of Newcastle Dental School, to speak at an ISRF symposium in London in June 1969 [51]. Jenkins’s assessment of research on interventions that reduced the harm of sugar consumption without restricting intake (Table 3, column A) was largely unfavorable [51]. Jenkins reviewed food additives, which in preliminary studies reduced the yield of bacterial acid produced after sugar consumption, and concluded that the dose of additives needed might be so high as to render the methods impractical or cause harmful side effects [51]. Perhaps unaware that ISRF was supporting research on dextranase and a tooth decay vaccine at the time under Project 269, Jenkins expressed skepticism about these lines of research:

Several lines of evidence have tended to emphasize, and perhaps exaggerate, the importance of dextrans.…As an enzyme its instability would limit its application, and the whole basis of this idea depends on the unresolved question of the importance of dextrans. [51]

On the caries vaccine Jenkins noted, that while “a successful preliminary experiment along these lines has been reported in three monkeys,” the promise of this result was limited because “it is admitted that the organisms used in the above experiment would be unsuitable for human use and it is not yet possible to incriminate any individual species [of bacteria] as the sole cause of human caries” [51]. Jenkins saw fluoridation as “the only thoroughly well-established method of reducing caries which does not require the active (and usually reluctant) participation of the patient” [51].

#### Industry receives a preview of the NIDR’s research priorities

ISRF got a preview of the NIDR’s research priorities for the NCP at the second ISRF symposium in September 1969 in Bethesda [52]. Richard Greulich, the NIDR’s intramural scientific director [31], spoke on “The Future of Caries Control” one month before the NIDR Caries Task Force Steering Committee would first discuss NCP research priorities (Table 1) [52]. Greulich said that while water fluoridation (which had been accepted in the US in 1965 as a “proved highly beneficial public health measure ready for widespread implementation” [29]) had achieved some success, The NIDR knew it was not the sole answer to eradicating dental caries:

From a public health point of view, we do not feel confident that fluoride is the only answer; and biologically speaking, it obviously is not because we have not talked to the other enterprises here. We have mentioned a host factor as represented or reflected by fluoridation. We have not talked to the microbes; we have not talked to the substrate or to nutrition. [52]

Greulich’s symposium presentation downplayed the value of limiting sucrose consumption as a means to control dental caries:

One could say, on logical grounds and good evidence, that if we could eliminate the consumption of sucrose, we could eliminate the problem—because we would be denying these pathogens their primary source of nutrient. We are realists, however, and we recognize the value of sucrose to nutrition. So *while it is theoretically possible to take this approach to demonstrate it*, *and it has been demonstrated certainly in animal models*, *it is not practical as a public health measure*. It is like saying the maximum speed of a jet plane is the speed of light. It just is not practical to try and evolve on to that point. And so in smooth surface caries, we have a more practical goal in working on the microorganism. [52] (emphasis added)

Similar to the approaches the sugar industry was promoting, Greulich identified interventions targeting bacteria as promising to the NIDR (Table 3, column B), including dextranases, for which the NIDR had been working with the pharmaceutical company Merck Sharpe & Dohme to think through the steps necessary for practical application [52]. The NIDR was also hopeful about a laboratory finding on “low molecular weight dextrans,” another substance that might be delivered to keep bacteria from producing harmful acid when exposed to sugar [52].

Beyond its focus on decay-causing bacteria, Greulich told ISRF that the NIDR was investigating ways to modify sugar to reduce its harmful effects [52]. These dietary modification interventions included adding phosphates to sugar, and the possibility of replacing table sugar, in the form of sucrose, with a liquid sugar, that split the sucrose molecules into glucose and fructose, which were thought to be less harmful to teeth [47]. Just before concluding, Greulich again assured ISRF that the NIDR research was not a threat to sugar consumption: “I reiterate that the role of sucrose [in dental caries] is undeniable, yet there is very little that anyone would want to do about this other than to explore some of these possible [dietary] modifications” [52].

#### Industry convenes a panel that includes many members of the NIDR Caries Task Force

In October 1969, the NIDR Caries Task Force Steering Committee met to identify research priorities [29]. As Greulich predicted, the main approaches reviewed focused on interfering with bacteria and dietary modification of sugar (Table 3, column C) [29]. However, a summary of the Caries Task Force Steering Committee meeting indicates that they “also reviewed the agenda for a conference on the role of human foodstuffs in dental caries” [29]. Caries Task Force Steering Committee member Basil Bibby would participate in the conference organization [42], and would have the chance to discuss the state of research on models identifying the cariogenicity of foods with the Caries Task Force, but not until March 1970 [43].

In October 1969, the same month the Caries Task Force Steering Committee was evaluating research priorities to eradicate dental caries (Table 1) [31,71], ISRF President Ross convened his Panel Meeting of the Dental Caries Task Force to consult on ISRF’s dental caries research priorities [53]. As Table 2 illustrates, the membership of ISRF’s panel overlapped almost completely with the NIDR Caries Task Force Steering Committee. All members of the NIDR Caries Task Force Steering Committee sat on the ISRF expert panel, with the exception of Fitzgerald, whose research on *S*. *mutans* had identified sucrose as the worst offender in smooth surface cavities [31,53]. The significant overlap between the membership of the ISRF expert panel and that of the NIDR Caries Task Force Steering Committee gave ISRF direct access to the NIDR’s Caries Task Force Steering Committee.

ISRF’s summary of the ISRF Panel Meeting of the Dental Caries Task Force indicates that the ISRF panel “recommended that a study be made of the cariogenicity of carbohydrate-containing foodstuffs” but did not mention studying the tooth-decay-causing potential of foods in its final “major approaches to caries” [71] (Table 3, column D).

#### Industry submits recommendations to the NIDR

ISRF submitted the findings from its series of meetings to the NIDR Caries Task Force late in 1969 in a report titled *Dental Caries Research—1969* [46]. While recognizing the causative role of sugar in tooth decay, ISRF downplayed the feasibility of restricting consumption of sugars while promoting advances made in areas of dextranase and caries vaccine research [46]. It also summarized dental caries interventions that would reduce the harm of sugar without impacting consumption, including phosphate food additives, protective sealants, and fluoride delivery through expanded community water programs, topical application, and addition to sugar, salt, or flour [46]. The research priorities identified by the NIDR Caries Task Force Steering Committee in October 1969 (Table 3, column C) are strongly aligned with ISRF’s submission (Table 3, column E), with the notable exception of developing a model to identify the cariogenicity of foods.

During fall 1969, the Nixon administration focused on biomedical research policy and showed signs of interest in supporting the NCP [31]. In January 1970, Caries Task Force Chairman Scherp submitted the report *A National Caries Program of the National Institute of Dental Research*: *Ten-Year Program of Research and Development* [41] in response to a request from the Office of the Secretary of Health, Education, and Welfare for a detailed plan for developing dental caries interventions [31]. Scherp’s report was based on the work of the NIDR Caries Task Force Steering Committee at its October meeting [31]. Later that month, the Assistant Secretary for Health indicated that President Nixon would endorse the program [31].

### Launch of the National Dental Caries Program, 1970–1971

During his February 1970 budget message, President Nixon announced support for “substantial increases in research on cancer, heart disease, serious childhood illnesses, and dental health—where current findings promise significant advances for the future” [31]. A line item in the budget allocated US$5 million (US$30.6 million in 2014 dollars) for the NCP in fiscal year 1971 [29].

In February 1970, after President Nixon’s public endorsement of the NCP but before the NIDR officially released the NCP research priorities, ISRF mailed its report *International Sugar Research Foundation Special Report*: *Dental Caries* [50] to its Scientific Advisory Board. The ISRF report began, “The correlation between sugar and dental decay—a practical concern of the sugar industry for many years—may become a purely academic issue within the foreseeable future,” then described the work ISRF leaders had invested to influence the NCP [50]. ISRF President Ross had collaborated with the NIDR Caries Task Force Chairman Scherp and had submitted a report created by ISRF staff on dental caries research priorities directly to the NIDR Caries Task Force:

Dental caries has been a constant worry to many consumers of sugar and sugar products. To some scientists, dental caries and sugar are considered almost “synonymous.” ISRF, in its concern about this image, has supported research to uncover many of the unknowns, and has kept in close communication with other institutions which concentrate on such research. The National Institute of Dental Research, of the U.S. Public Health Service’s National Institutes of Health, is the most prominent U. S. organization conducting dental caries research on a broad scale. Last year the Institute formed a Dental Caries Task Force to work “toward the goal of virtually eliminating tooth decay in the United States.” Dr. Philip Ross, ISRF President, met with the Dental Caries Task Force and has worked closely with its Chairman, Dr. Henry W. Scherp. Dental Caries Research—1969, prepared several months ago by the staff of ISRF, reviewed current knowledge of the subject and was submitted to the Task Force for its consideration. [50]

The NIDR Caries Task Force held its conference on dietary research priorities one month later (Table 1) [42]. At the NIDR Role of Human Foodstuffs in Caries Workshop Conference, Caries Task Force Steering Committee member Basil Bibby presented a paper, “Methods for Comparing the Cariogenicity of Foodstuffs,” which reviewed the status of research on experimental models to identify food products harmful to teeth [43]. These models were important, according to Bibby, because it was “desirable to have a relatively speedy and economical method of evaluating cariogenicity, especially of snack-type foods, so that parents can be warned against the more destructive products” [43]. Bibby’s presentation summarized 12 different models to identify the cariogenicity of foods, ranging from “acid production from foods incubated in saliva” to the production of caries in rats, monkeys, and pigs [43]. During the discussion of Bibby’s presentation, Caries Task Force members established that “a quick screening method was needed to provide presumptive evidence of the potential cariogenicity of accepted foods and new products that appear almost daily on the shelves of food markets,” although there were differences of opinion on what the best model would be to screen for cariogenicity [44]. No one argued that the NIDR not pursue standardization of a test that would rank foods on their potential for tooth decay [44].

### Comparison of ISRF and the NIDR Research Priorities

Soon after Nixon’s February 1970 endorsement of the NCP, Scherp began operational planning for program implementation at the NIDR [29]. Research priorities were first published in an omnibus request for contracts (RFC) [29] titled *Opportunities for Participation in the National Caries Program* [30] in early 1971. The NIDR received 112 proposals and funded 17 contracts [29] totaling US$3 million (US$18.3 million in 2014 dollars) out of the NCP’s budget of US$6 million (US$36.7 million in 2014 dollars) [31]. While the 1971 NCP RFC was the first of several RFCs [73], it established the NIDR’s research priorities for years [29].

The research priorities in the 1971 NCP RFC largely reflected the research priorities identified at the October 1969 NIDR Caries Task Force Steering Committee meeting (compare columns C and G in Table 3). Despite being published nearly a year after the NIDR Caries Task Force Role of Human Foodstuffs in Caries Workshop Conference (Table 1), the 1971 NIDR RFC omitted developing a standardized model to identify the cariogenicity of foods as a research priority.

Comparison of the research priorities identified by ISRF and submitted to the NIDR in 1969 (Table 3, column E) with those published by the NIDR in its 1971 NCP RFC (column G) shows that ISRF and the NIDR research priorities were largely aligned. Indeed, a side-by-side comparison of overlapping text from the ISRF submission to the NIDR, *Dental Caries Research—1969* [46], and the 1971 NCP RFC, *Opportunities for Participation in the National Caries Program* [30], reveals that 78% of the ISRF submission to the NIDR was directly incorporated into the 1971 NCP RFC. (S1 Table provides the actual text from the ISRF submission and 1971 NCP RFC.) Of the 274 total lines in the 1971 NCP RFC describing research priorities, 110 lines, or 40%, were taken verbatim or closely paraphrased from the ISRF submission. Of these 110 lines, 34% were copied verbatim from the ISRF report, and 66% were paraphrased.

## Discussion

This study analyzes a series of papers discussing previously undocumented cane and beet sugar industry activities between 1959 and 1971 regarding strategies to influence the research priorities of the NIDR’s 1971 NCP. The documents show that the sugar industry knew that sugar caused dental caries as early as 1950 and did not attempt to deny the causative role of sucrose in tooth decay. Instead, through trade associations, the sugar industry adopted a strategy to deflect attention to public health interventions that would reduce the harm of sugar consumption, rather than restricting intake.

After the NIDR announced it was considering a research program to eradicate dental caries in 1966, the sugar industry used tactics designed to protect sucrose sales. In collaboration with the chocolate and confectionary industries, SRF funded research that supported the idea that enzymes and a tooth decay vaccine could be developed that could eradicate dental decay without requiring sugar restrictions. ISRF conducted reviews of the dental caries literature to identify potential interventions that might reduce the health harms of sugar consumption other than by restricting sugar intake. ISRF cultivated relationships with the NIDR leadership through meetings with the Caries Task Force chairman and through a consultation with members of the NCP steering committee charged with selecting research priorities. A sugar industry report submitted to the NIDR became the basis for the research priorities published in the first NCP RFC.

While not officially recognized as participating in the NIDR Caries Task Force, the sugar industry effectively contributed to the research priorities developed for the launch of the NCP. Research priorities identified in the first NIDR NCP RFC focused on sugar harm reduction strategies, as opposed to sugar restriction, and were strongly aligned with sugar industry research priorities. The NIDR, like ISRF, took the position that sugar restriction was impractical.

The first policies related to the declaration of conflicts of interest for federal advisory committees were implemented in the early 1960s [74]. Prior to that, concern that industry interests were a threat to scientific integrity was not a majority view [75]. Significant consumer concern about corporate influence on expert committees would not surface until the 1970s, after the launch of the NCP. By contrast, in 2015, the NIH had an entire program dedicated to ethical contact within its institutes [76] because of the greater awareness of industry conflicts of interest and how they can adversely impact the scientific enterprise.

### The 1970s Missed Opportunity

The majority of the research priorities promoted by the sugar industry and those selected for the 1971 NCP RFC failed to lead to widespread application [31]. By 1976, clinical studies of dextranase mouth rinses in humans had failed to duplicate the success of using dextranases to inhibit new dental caries in experimental animals [31]. The NIDR found that the pharmaceutical industry had limited interest in research, development, and distribution of antimicrobial agents, because of the high cost of regulatory approval by the Food and Drug Administration (FDA) and doubts about identifying an agent that would be successful on a large scale [31]. By 1977, NCP researchers had found that their plan to substitute sucrose with a mixture of glucose and fructose “would effect little reduction in food cariogenicity” [29]. In addition, by 1978, the NIDR had terminated clinical trials on phosphates added to foods because they were ineffective [31].

The most successful interventions selected for funding following the 1971 NCP RFP were topical fluoride and sealants [31]. While a 1980 prevalence survey found that the burden of dental disease in children had decreased by more than 30% since the last survey in 1971–1973, 64% of children still exhibited dental caries, far short of the NCP’s founding goal of eradicating the disease [31].

It is not clear why the NIDR adopted the position in 1969 that reducing sugar intake as a public health measure was impractical. Proposals centered on ways to limit sucrose consumption were just around the corner. In its multi-year review of foods generally recognized as safe initiated in 1969, the FDA deemed sucrose consumption at 1976 levels as unsafe for teeth [77]. In the coming years, the FDA would consider food labels “to warn against the hazards to the teeth of consuming a particular product” and debate whether warning labels should be placed on foods based on the percentage of sugar content, or on some measure of cariogenic potential [78].

When reflecting on the NCP in 1990, Basil Bibby, a member of the Caries Task Force Steering Committee, noted that the NIDR approved only “one or two small research grants” related to food cariogenicity compared to the “hundreds of generous awards [that] were made for investigations with so-called high scientific content” [79]. He also noted that since the NIDR was the major funding source for dental research in the US, “the failure of the National Institute for Dental Research to support research on foods meant that there was no group of investigators in the United States who had enough financial support to undertake significant research on food cariogenicity” [79].

In 1977, the NIDR finally moved to develop a standardized animal model to identify the tooth-decay-causing potential of foods “with the objective of its being widely accepted in industry, and in regulatory agencies and in academic research, as a basis for distinguishing cariogenic from non-cariogenic snacks” [29]. While research on an animal model was initiated at the NIDR [29], the bulk of the research was conducted outside the NIDR, largely funded by the American Dental Association Health Foundation [80]. Based on the promise of the development of a standardized model to identify harmful foods, in 1978 the US Federal Trade Commission proposed restrictions on advertising cariogenic products to children [81]. The first US Department of Health and Human Services Healthy People objectives, issued in 1980, proposed banning cariogenic products from schools as a means to control dental caries [82]. While lobbying efforts of the food, advertising, and broadcasting industries were a major reason for the failure of the FDA, Federal Trade Commission, and Healthy People proposals, another common factor cited for these policy failures is the lack of a standardized model to identify foods harmful to teeth [78,81,83].

With industry input, consensus was finally achieved on a standard method to screen foods for cariogenicity at a conference sponsored by the Foods, Nutrition and Dental Health Program of the American Dental Association in 1985, but only to support claims that food products were safe for teeth [84]. In 1996, the FDA began allowing health claims (i.e., “does not promote tooth decay”) on food products containing sugar substitutes based on a standard screening method for cariogenicity [85]. The FDA did not, however, require disclosure or labeling of harmful foods. In 1999, a group of clinicians and dental scientists updated the methodology agreed upon in 1985 with the aim of identifying which methods were “suitable as research tools but also for regulatory assessments” [86]. However, the use of these methods to identify foods harmful to teeth remained controversial [87].

With the implementation of the nutrition facts panel on packaged food products in 1993, the FDA required the declaration of total sugars [88], a requirement that remained unchanged as of January 2015. As of January 2015, the FDA was considering a proposed rule to require disclosure of added sugars on the nutrition facts panel [88], and SA was opposing it, citing “the lack of science to justify ‘added sugars’ labeling” [89].

### Comparison to the Tobacco Industry

The sugar industry formed SRF in 1943 to fund research that supported the industry position [34], 11 years before the creation of the Tobacco Industry Research Committee (TIRC) in 1954 to play a similar role for the tobacco industry [90]. In 1954, the TIRC hired SRF’s first scientific director, Robert Hockett, to serve as the TIRC’s associate scientific director [91], where he was positioned to help the tobacco industry learn key science manipulation tactics from the sugar industry.

At the same time that the NIDR was planning the NCP, the National Cancer Institute (NCI) was pursuing its Smoking and Health Program [92–94]. Like NCP, which focused on sugar harm reduction strategies, the Smoking and Health Program focused on harm reduction strategies with the primary goal of developing a safe cigarette [93]. The NCI invited tobacco industry representatives to join the NCI’s Tobacco Working Group (TWG), the planning committee for the effort to develop a less hazardous cigarette [93]. The NCI did so on the assumption that tobacco manufacturers were interested in promoting new, safer cigarettes and had product expertise the NCI lacked [94]. The NCI also believed industry participation was advantageous because implementation would fall to tobacco companies and, if approached in a positive way, the companies would agree to collaborate [94]. The willingness of the NIDR leaders to interact with the sugar industry during planning for the NCP may have reflected similar thinking, particularly because responsibility for manufacturing and incorporating additives to reduce the risk of dental caries would fall to food and pharmaceutical industries.

The tobacco industry used its involvement in the TWG to oppose funding of projects, such as smoking cessation programs, that were seen as a threat to industry interests [94]. The tobacco industry also withheld knowledge about the biological effects of cigarette smoke and human smoking behavior, which negatively impacted the NCI’s efforts [94]. Indeed, industry use of the TWG to block effective tobacco control strategies was cited by federal Judge Gladys Kessler in her 2006 ruling that the major cigarette companies and their research and lobbying organizations had formed an illegal enterprise to defraud the public in violation of the Racketeer Influenced and Corrupt Organizations Act [95].

Litigation against tobacco companies has been a major factor in achieving meaningful policy change. Successful litigation could not have been achieved without industry documents research illuminating the strategies and tactics of tobacco companies. This analysis demonstrates that sugar industry documents research has the potential to define industry strategies and tactics, which may potentially prove useful in future litigation.

### Limitations

While we were fortunate to discover the Roger Adams papers, we recognize that it provides a narrow window into the activities of just one sugar industry trade association, particularly because other industries had an interest in the outcome of the NCP, including the chocolate and confectionary industries, the pharmaceutical industry, and food companies interested in developing food additives and sugar substitutes. To help compensate for limited access to industry documents, we used other historical materials to cross-validate findings as they emerged throughout the analysis. Another limitation was that we could not interview key actors.

### Conclusion

This historical example illustrates how industry protects itself from potentially damaging research, which can inform policy makers today. While it may be valuable in theory for the industry to contribute data about their products to the research community, industry should not have the opportunity to influence public health research priorities [94]. Regulatory science to support sensible and defensible policies to limit added sugar consumption was not pursued in the 1970s because of the alignment of the NIDR’s research priorities with those of the sugar industry. Actions taken by the sugar industry to impact the NIDR’s NCP research priorities, which echo those of the tobacco industry, should be a warning to the public health community. The sugar industry’s current position—that public health recommendations to reduce dental caries risk should focus on sugar harm reduction as opposed to sugar restrictions—is grounded in more than 60 years of protecting industry interests. Industry opposition to current policy proposals—including a WHO guideline on sugars proposed in 2014 and changes to the nutrition facts panel proposed in 2014 by the FDA—should be carefully scrutinized to ensure that industry interests do not supersede public health goals.

## Supporting Information

S1 Table Comparison of ISRF’s submission to the NIDR Caries Task Force, *Dental Caries Research—1969*, to NIDR’s 1971 National Caries Program request for contracts, *Opportunities for Participation in the National Caries Program*. (PDF)

## Abbreviations

FAO: Food and Agriculture Organization
FDA: Food and Drug Administration
ISRF: International Sugar Research Foundation
NCI: National Cancer Institute
NCP: National Caries Program
NIDR: National Institute of Dental Research
NIDCR: National Institute of Dental and Craniofacial Research
NIH: National Institutes of Health
RFC: request for contracts
SA: the Sugar Association
SRF: Sugar Research Foundation
TIRC: Tobacco Industry Research Committee
TWG: Tobacco Working Group
WHO: World Health Organization
WSRO: World Sugar Research Organisation
