# Supplementary material for: Sugar Industry Influence on the Scientific Agenda of the National Institute of Dental Research’s 1971 National Caries Program: A Historical Analysis of Internal Documents
Source: PLoS Med. 2015 Mar 10;12(3):e1001798. doi: 10.1371/journal.pmed.1001798 (PMC4355299; doi:10.1371/journal.pmed.1001798)
Supplement: S1 Table — (PDF) [file pmed.1001798.s001.pdf]

Sugar Industry Influence on the Scientific Agenda of the National Institute of Dental Research's 1971 National Caries Program: A Historical Analysis of Internal Documents

Supplemental Table

Cristin E. Kearns, DDS, MBA
Stanton A. Glantz, PhD
Laura A. Schmidt, PhD, MSW, MPH

Table S1: Comparison of ISRF's submission to the NIDR Caries Task Force: *Dental Caries Research--1969* to NIDR's 1971 National Caries Program request for contracts, *Opportunities for Participation in the National Caries Program.* (Text from both documents is contiguous. Verbatim text is bolded.)

**ISRF 1969 Submission to NIDR** [1]	**NIDR 1971 National Caries Program RFC** [2]
**Dental caries** may be described as a **localized progressive**, molecular disintegration of tooth structure. It is thought to be **the most prevalent** disease affecting mankind.<br>Although much has been and is being done to combat it, some 90% of the people in the world experience dental caries. In the United States the incidence is nearer 98%.<br>What causes this disease? What are the prospects for its eradication or control?<br>Research has shown that the development of caries requires interactions between tooth surface, oral bacteria, and dietary carbohydrate. Although basically a microbial disease, it is nonetheless influenced greatly by such factors as genetics, age, diet, nutrition, environment, and oral hygiene. The process begins when oral bacteria establish themselves on the teeth in a sticky plaque which adheres to the enamel surface. Decay action occurs as a result of bacterial fermentation of **dietary carbohydrate principally to lactic acid which, at susceptible sites, initiates a carious lesion by demineralizing the enamel surface**. **The predominant group of cariogenic bacteria metabolizes sucrose** in a peculiar way, **producing** an **adhesive polysaccharide (dextran)** from the glucose factor and lactic acid from the fructose factor. **Typically, these bacteria also store intracellular polysaccharide (amylopectin)** during periods of environmental carbohydrate abundance **and utilize it with the formation of lactic acid** during periods of environmental carbohydrate deficiency.<br>Because **the development of caries requires** critical relationships between **tooth surface**, **oral** bacteria, **and dietary carbohydrate**, the means to **control** the disease should be found in a modification **of one or more of these three factors**. With no lead that promises to do more than	INTRODUCTION<br>**Dental caries** is **localized**, **progressive** decay of the teeth. It is initiated by demineralization of the surface of the tooth by organic acids produced locally by bacteria that ferment deposits of carbohydrate foods. With progressive loss of tooth mineral and secondary destruction of tooth protein by continued bacterial action, cavities form. These, if untreated, extend and destroy most of the tooth, often leading to serious infection of the surrounding tissues. Almost everyone in the United States experiences dental caries to some degree, mostly before adulthood. This disease is the leading cause of lost teeth before age 35, when chronic progressive destructive periodontitis (pyorrhea) begins to supervene. Though not ordinarily considered to be life endangering, these two diseases are among **the most prevalent** and troublesome afflictions of man.<br>In the United States it has been estimated that about $2,000,000,000 is spent annually to repair the resultant damage of tooth decay. Even so, we meet only a minor fraction of the need. Since caries is principally a disease of young people, a recent study by the United States Army gives a representative picture of the problem. A survey of men at induction centers over a one and one half year period showed the treatment requirements for each 1,000 men: operative dentistry-8,500 surfaces; extractions-1,008; crowns, partial or complete prostheses-794. A similar survey of the dental needs of 1,500 U.S. Marine recruits showed similar findings (per 1,000 men): restorations-5,050; extractions-511. It is estimated that to repair completely the damage caused by caries nationwide would cost $8,000,000,000 more annually than we now spend.<br>Review of the caries research already accomplished warrants the expectation that these

**Table S1: Comparison of ISRF's submission to the NIDR Caries Task Force: *Dental Caries Research--1969* to NIDR's 1971 National Caries Program request for contracts, *Opportunities for Participation in the National Caries Program*. (Text from both documents is contiguous. Verbatim text is bolded.)**	
**ISRF 1969 Submission to NIDR** [1]	**NIDR 1971 National Caries Program RFC** [2]
arrest a carious lesion once it is clinically detectable, priority should be given to research that will provide preventive control.	deplorable statistics could be greatly reduced. During the past decade, dental caries research has experienced an impressive upsurge, catalyzed primarily by experimental substantiation of the concept that caries results from one or more transmissible infective agents. Specifically, caries results from colonization of vulnerable surfaces of the teeth by a characteristic group of bacteria. These bacteria ferment **dietary carbohydrates** in situ, **principally to lactic acid, which at susceptible sites, initiates the carious lesion by demineralizing the enamel surface. The predominant group of cariogenic bacteria metabolize sucrose**, **producing** extracellularly **an adhesive polysaccharide (dextran)**. **Typically, these bacteria also store intracellular polysaccharide (amylopectin) and utilize it with the formation of lactic acid. The development of caries requires** a susceptible **tooth surface**, **oral** microbiota, **and dietary carbohydrate**. The logical approach to control therefore, is to modify one or more of the three factors in this host-parasite environment complex.<br>GENERAL PROGRAM OBJECTIVES<br>By a concerted effort to apply existing knowledge, to follow established leads, and to foster the fundamental research judged most likely to produce utilizable new information it is theoretically possible to prevent dental caries. To this end the National Institute of Dental Research has embarked in a National Caries Program, guided by an advisory committee of leading scientists representing the various phases of caries research and drawn from both within and without the Institute.<br>The purpose of the lnstitute's program is to reduce the incidence of caries and to extend the capabilities of the dentist, the hygienist, and others on the dental team to prevent decay. Because of the complex nature of caries, it is unlikely that any one approach will completely solve the problems of its prevention and control. Efforts are therefore directed to depressing the effects of all factors to a minimum and utilizing a combination of techniques instead of concentrating on one.<br>In seeking areas where results are likely to benefit the most people promptly, three questions must be asked: What measures of proved efficacy are being used inadequately? What measures have

Table S1: Comparison of ISRF's submission to the NIDR Caries Task Force: *Dental Caries Research--1969* to NIDR's 1971 National Caries Program request for contracts, *Opportunities for Participation in the National Caries Program.* (Text from both documents is contiguous. Verbatim text is bolded.)	
**ISRF 1969 Submission to NIDR** [1]	**NIDR 1971 National Caries Program RFC** [2]
	been sufficiently proved by preliminary clinical trials to warrant large-scale field demonstration or national application? What fundamental research is ready for intensive development and clinical trial?<br>PROGRAM EMPHASIS<br>At present there are in view no therapeutic methods that do more than arrest clinically detectable carious lesions. For control of caries emphasis must be placed on prevention. Experience cautions us, however, that prevention will be achieved only gradually. Therefore, heavy demand for restorative dentistry will continue and so will the need for improved restorative materials and procedures. This includes replacement of lost teeth with natural teeth or with synthetic substitutes, to restore both function and esthetic appearance. Work along this line is encouraged although it is not anticipated that this will be an area of major investment.<br>Dental caries is a disease which develops slowly. It is essential to develop caries-susceptibility tests and procedures for shortening the present two-to-three year time needed for evaluation of anticaries measures. Investigations along this line are particularly encouraged.<br>Promising leads, some of which are described later in this brochure, will be pursued through an appropriate sequence of studies: laboratory research, clinical studies, field trials and field demonstrations. When they reach the point of readiness, they will be applied widely in personal oral health programs and/or in community health services. The various research leads which are mentioned herein are given to illustrate the multifaceted program which is anticipated. The alternatives which may be pursued are unlimited, and are restricted only in that they meet the goal of the Program: prevention and control of dental caries.
At present, **adequate** intake **of fluoride remains the one proved means to increasing the resistance of teeth to caries**. **The experience of 25 years leaves no doubt that a daily intake throughout life of about one milligram of fluoride per person, as commonly provided by from 0.7 to 1.0 part per million in the water**	DENTAL CARIES<br>Protecting the Teeth<br>Fluoride<br>**Adequate** incorporation **of fluoride** in teeth, particularly in the outer layers of the enamel, **remains the one** thoroughly **proved means to increase resistance of teeth to caries**. **The experience of 25 years leaves no doubt that a daily intake throughout life of about 1 mg of fluoride per person, as commonly provided by**

Table S1: Comparison of ISRF's submission to the NIDR Caries Task Force: *Dental Caries Research--1969* to NIDR's 1971 National Caries Program request for contracts, *Opportunities for Participation in the National Caries Program*. (Text from both documents is contiguous. Verbatim text is bolded.)

**ISRF 1969 Submission to NIDR** [1]	**NIDR 1971 National Caries Program RFC** [2]
**supply, harmlessly lowers the caries rate by 50 to 60 per cent in permanent teeth and slightly less in deciduous teeth**, under present conditions in the United States.<br>According to the 1967 Fluoridation Census, only 52.8 per cent of the U. S. population using public water supplies is receiving this benefit, 46.3 per.cent by controlled artificial fluoridation and 6.5 per cent by natural fluoridation. **Twenty-two per cent of the total U. S. population (44,000,000 persons)** are **not** on **public water systems**, **though presumably many ingest naturally fluoridated water and others receive controlled amounts of fluoride by other means**. **Clearly a major effort is needed to fluoridate more communal water supplies and, by alternate means, to get fluoride to the large fraction of** persons **not accessible** at present. The latter group might be reached, by diet, as by the addition of fluoride to sugar, salt, flour or other widely consumed ingredient; by direct ingestion of fluoride tablets or solutions; or by do-it-yourself topical application of fluoride.<br>**What about the enormous number of carious lesions that develop despite fluoridation?** Is the current **dosage o**f **fluoride sufficient**? The thorough epidemiological studies of the past indicated that more than one part per million of fluoride in the water supply did not confer much additional protection against caries in permanent teeth. Some investigators have recommended two parts per million as more beneficial for deciduous teeth, though at some risk of moderate fluorosis or mottling of the enamel, in permanent teeth. **Recent studies**, however, suggest **that topical application of fluoride**, **to increase the fluoride content of the outer few microns of enamel** above the **level acquired from fluoridated water**, might **reduce caries** incidence **by** an additional 20 to 30 **per cent**, with little or no risk of dental fluorosis. If this measure proves to be as effective as current clinical trials indicate, wide application would be well worthwhile.	**from 0.7 to 1.0 ppm of fluoride in public water supplies, harmlessly lowers the caries rate by from 50 to 60 percent in permanent teeth and slightly less in deciduous teeth**. Logically, a national program to prevent caries should be based on universal fluoridation. **Twenty-two percent of the total U.S. population, or 44,000,000 persons**, do **not** have access to **public water systems**, **though presumably many ingest naturally fluoridated water and some receive controlled amounts of fluoride by other means**. **Clearly a major effort is still needed to fluoridate more communal water supplies and by alternate means to get fluoride to the large fraction of** our population **not** thus **accessible**.<br>**What about the enormous number of carious lesions that develop despite fluoridation?** Are we recommending a sufficient **dosage of fluoride**? **Recent studies** indicate **that** intensive **topical application of fluoride, to increase the fluoride content of the outer few microns of enamel** to two or three times the average **level acquired from fluoridated water**, can **reduce caries by** as much as 75 to 80 **percent**, that is, half again as much reduction as effected by controlled fluoridation of water supplies.<br>Answers are being sought to these questions:<br>1. What level of enamel fluoride provides optimum protection against caries?<br>2. What is the most rapid and efficient method of achieving this level?<br>3. What supplemental applications are required to maintain this level?<br>More knowledge also is required to fully understand the action of fluorides on solubility of tooth enamel, on remineralization of the tooth surface, and on bacteria and their products. The effect of fluoride on decay-causing organisms and their metabolic by-products must be investigated further in order to obtain clues for developing methods of reducing their cariogenicity.
**Caries that develop despite fluoride occur principally in pits and fissures on the occlusal**, or biting, **surfaces of** teeth. Prevention by **sealing** these **surfaces with** a durable **adhesive** material has been shown to be feasible, though not yet fully practicable. Current investigations promise to develop more serviceable materials.	<u>Sealants</u><br>**Caries that develops despite optimal fluoridation of teeth occurs principally in the pits and fissures** that are a normal feature of **the occlusal surfaces** of the molars and bicuspids. This is usually attributed to impaction of food residues and bacteria plus thinness of the enamel in these areas. Newly erupted teeth are the most

**Table S1: Comparison of ISRF's submission to the NIDR Caries Task Force: *Dental Caries Research--1969* to NIDR's 1971 National Caries Program request for contracts, *Opportunities for Participation in the National Caries Program.* (Text from both documents is contiguous. Verbatim text is bolded.)**	
**ISRF 1969 Submission to NIDR** [1]	**NIDR 1971 National Caries Program RFC** [2]
	vulnerable. It was shown in the 1920's that these pit-and-fissure areas could be protected against caries either by grinding them out to form wide nonretentive grooves or by filling them with dental amalgam.<br>These procedures, however, did not gain wide popularity. Now it is believed that the same result can be accomplished **by sealing** the occlusal **surfaces with** an **adhesive** polymer. Preliminary results show that treated sites developed no caries whereas 42 percent of an equal number of untreated sites became carious during a two-year period. Occlusal surfaces possibly can be sealed soon after eruption of the tooth, to protect them during their most caries-susceptible period. It will be necessary, however, to answer the question: does early sealing impede the normal maturation of a tooth thereby leaving the pits and fissures indefinitely susceptible to caries, if uncovered?<br>Other questions requiring answers are:<br>1. Can the use of sealants be effectively coupled with topical fluoride treatments?<br>2. Are there other materials that can be more easily and effectively used on the tooth surface for sealing purposes?<br>3. Can sealants be effectively applied to areas of the teeth other than occlusal surfaces?
As to diet, **an abundance of epidemiological and experimental evidence** shows **that sucrose is** a particularly **cariogenic** culprit **in our modern diet**. So far as we know, this unfortunate property relates to the peculiar way in which sucrose is metabolized by cariogenic streptococci. Many dental research scientists feel that if people were to get practically all of their carbohydrate from starchy foods, and if there were adequate fluoridation, coronal caries, or caries occurring in the exposed portion of teeth, would almost certainly be negligible. Such is the case in regions of Southeast Asia, for example. Whether replacement of dietary sucrose by other sugars, rather than by starch, would reduce human caries as effectively has not been ascertained. In animal studies, however, such sugars as **glucose** and **fructose have** on the whole **induced** strikingly **less** incidence of **caries than sucrose**. But **replacement of sucrose**, the universal	<u>Modifying the Diet</u><br><u>Sugar Substitutes</u><br>**An abundance of epidemiological and experimental evidence** indicates **that sucrose is** the principal **cariogenic** agent **in our modern diet**. In experimental caries in hamsters and rats, **glucose** or **fructose**, have generally **induced** much **less caries than sucrose**. Whether replacement of dietary sucrose by other sugars would reduce human caries has not been ascertained--there are no data.<br>In animal experiments the reductions in caries activity have been most pronounced on smooth surfaces of teeth, where development of caries seems to depend on <u>Streptococcus</u> <u>mutans</u> and its adhesion by extracellular dextran produced from sucrose. In the hamster, all caries is of this type because of the morphology of the teeth. In the deep fissures of the rat molars, on the other hand, food impaction makes adhesion unnecessary and indigenous acidogens, as well as

Table S1: Comparison of ISRF's submission to the NIDR Caries Task Force: *Dental Caries Research--1969* to NIDR's 1971 National Caries Program request for contracts, *Opportunities for Participation in the National Caries Program.* (Text from both documents is contiguous. Verbatim text is bolded.)

**ISRF 1969 Submission to NIDR** [1]	**NIDR 1971 National Caries Program RFC** [2]
natural food sweetener, **in** the **diet** is not a feasible solution. Many dental scientists feel, however, that if sucrose could be replaced by other sweets in candy alone, the results might be quite beneficial, judging by results with experimental caries. **Merely reducing the frequency of eating a high-sucrose diet significantly reduces caries-in rats.**	S. mutans, can initiate caries if provided with various fermentable sugars. Substitution of starch for sugars in animals, however, consistently reduces the caries scores to very low levels.<br>**Replacement of sucrose in** our **diet** would require quite a cultural and technological revolution, but might not be as impractical as it seems. Trials with candies made with a hydrogenated starch hydrolyzate have been made in Sweden. If sucrose could be replaced by other sweetening agents in candy or other between-meal snacks, the result might be quite beneficial. This, at any rate, is suggested by results from animal studies and epidemiological data from humans. **Merely reducing the frequency of eating a high-sucrose diet significantly reduces caries in rats.** In humans, increased frequency of between-meal eating of sugary snacks correlates with increased caries attack. This emphasizes the importance of keeping as low as possible the intraoral accumulation of sucrose, whether by reducing the frequency of intake, avoiding adherent sweetstuffs, or diluting the sucrose in sweetstuffs with other sweeteners.<br>Important problems in this area requiring resolution are:<br>1. Would replacement of sucrose in the diet of humans by other types of sugars effectively reduce caries?<br>2. Can sucrose substitutes be developed for use in the manufacture of confections, baked goods, and desserts?<br>3. Can the properties of sucrose in food be modified so that the foods are less cariogenic?
But since it is **not practicable to replace sucrose in our diet**, **can anything be added to mitigate its cariogenicity? Phosphates are a possible answer.** A plenitude of **laboratory studies** in rodents **agree that addition of any of a wide variety of inorganic and organic phosphates to high-sucrose and other cariogenic diets significantly reduces caries, in some experiments almost completely**. **Unfortunately, the relatively few clinical trials reported so far** have not yet established **unequivocally whether or not a phosphate supplement reduces caries in humans.**	Dietary Additives<br>If **it is not practicable to replace sucrose in our diet, can anything be added to the diet to mitigate its cariogenicity? Phosphates are a possible answer.** More than 150 **laboratory studies agree that addition of any of a wide variety of inorganic and organic phosphates to high-sucrose and other cariogenic diets significantly reduces caries** in rats and hamsters, **in some experiments almost completely**. So far, the cyclic condensed salt, sodium trimetaphosphate, has been the most effective one. How phosphates mitigate caries has not been ascertained, except that they act locally in the oral cavity and seem to benefit newly erupted teeth the

**Table S1: Comparison of ISRF's submission to the NIDR Caries Task Force: *Dental Caries Research--1969* to NIDR's 1971 National Caries Program request for contracts, *Opportunities for Participation in the National Caries Program*. (Text from both documents is contiguous. Verbatim text is bolded.)**	
**ISRF 1969 Submission to NIDR** [1]	**NIDR 1971 National Caries Program RFC** [2]
	most. Unfortunately, the relatively few clinical trials reported so far do not tell us unequivocally whether or not a phosphate dietary supplement reduces caries in humans. Translating the conditions of the animal model into a regimen suitable for delivering adequate extra phosphate to humans presents many complexities. Conceivably it might be helpful if a phosphate were incorporated in sweetened between-meal snacks alone. Also, since phosphates evidently prevent caries by local action in the oral cavity, frequent direct application of concentrated solutions to the teeth might be beneficial.<br>We would particularly like to know:<br>1. Will the incorporation of phosphates in different vehicles such as flour, salt, milk, or snack foods reduce the incidence of caries in humans?<br>2. Are there other dietary additives which could mitigate the cariogenic effects of sugar in the human diet?
	<u>Trace Elements</u><br>Epidemiologists have been struck by the wide variations in caries experience between different localities. These differences were greatest between low-fluoride areas, though they were discernible between high-fluoride areas also. It was suggested that caries resistance might be attributable not only to the fluoride content of drinking water but also to other elements found in such small quantities that they are known as trace elements. Only recently, however, has this problem begun to receive the epidemiological and laboratory study that it merits. One study has indicated a correlation between low caries experience and increased concentrations of boron, lithium, molybdenum, strontium, titanium, and vanadium in the drinking water. Attention to the mineral content of water alone, however, might mislead us. Information also is needed on the mineral content of the soil where foodstuffs are grown. Except for fluoride, available data indicate that from 80 to 90 percent of our trace element intake comes from foodstuffs. If correlations can be established between caries experience and these elements, and if a causal relation is found, an anticaries measure that will supplement controlled fluoridation could eventuate.<br>It is necessary for us to determine:<br>1. Are there trace elements other than

Table S1: Comparison of ISRF's submission to the NIDR Caries Task Force: *Dental Caries Research--1969* to NIDR's 1971 National Caries Program request for contracts, *Opportunities for Participation in the National Caries Program.* (Text from both documents is contiguous. Verbatim text is bolded.)

**ISRF 1969 Submission to NIDR** [1]	**NIDR 1971 National Caries Program RFC** [2]
	fluoride which are important in caries prevention?<br>2. Are there constituents of the water or soil products which accentuate the anticariogenic effect of fluorides?
The bacterial component of the carious complex in rats and hamsters comprises predominantly a group of anaerobic streptococci now being designated as Streptococcus mutans, a species that was first reported as the presumed cause of human caries 45 years ago but was not described adequately and was soon forgotten. **Streptococcal strains closely resembling the cariogenic S. mutans indigenous to rats and hamsters have been isolated regularly from human carious lesions**, and they induce caries when inoculated into germfree rats or suitable stocks of conventional rats and hamsters. The evidence for etiologic significance of S. mutans in human caries is therefore comparable to Koch's classic evidence for the causative role of the tubercle bacillus in tuberculosis in the last century.	Combatting Cariogenic Bacteria<br>While strong emphasis has been given to increasing the resistance of teeth to caries and to reducing the cariogenicity of the diet, proportionately little attention has been given to antimicrobial measures.<br>Oral infection with S. mutans and a diet high in sucrose are important and probably essential components for caries in hamsters, and for smooth-surface caries in rats. **Streptococcal strains closely resembling the cariogenic S. mutans indigenous to rats and hamsters have been isolated by direct culture regularly from human carious lesions**, where they frequently constitute the majority of the streptococci.<br>In addition to.S. mutans, some strains of several other bacterial species have induced coronal caries, when implanted in the oral cavity of experimental animals in conjunction with a high-sucrose diet. Included are strains of Streptococcus faecalis, Streptococcus sanguis, Streptococcus salivarius, streptococci not identifiable as recognized species, Lactobacillus acidophilus, and Lactobacillus casei. A preferential accumulation of lactobacilli, commonly in conjunction with streptococci has been demonstrated in dental plaque prior to caries, and also in carious lesions in humans and monkeys.
**Present evidence indicates that the greater cariogenicity of S. mutans, compared with a variety of other oral acidogens, relates to its characteristic of producing** from sucrose so-called **insoluble dextrans of high molecular weight.** These dextrans evidently are responsible **for the greater adhesiveness of** cariogenic strains of **S. mutans to the tooth surface.** This property suggested the possibility of anticaries measures directed against such dextrans. Thus, **incorporation of a dextranase preparation in the** diet and **drinking water**, or drinking water alone, dramatically **reduced** both **plaque** formation **and caries** in **hamsters on a high sucrose diet**. As a consequence of these experiments, purified and	Preventing Adhesion<br>A comprehensive program for preventing caries logically should include measures to reduce colonization of the teeth by cariogenic bacteria or to suppress their activities. These measures include mechanical cleansing, topical application of antibacterial agents, metabolic regulators to inhibit production of cariogenic products, enzymes to digest products conducing to adhesion of bacteria to teeth, and immunological measures. Alternatively, bacterial colonization might also be averted by chemically altering the enamel surface so that bacteria cannot adhere to it.<br>Numerous investigations during the past decade have substantiated the cariogenic importance of the anaerobic streptococci

**Table S1: Comparison of ISRF's submission to the NIDR Caries Task Force: *Dental Caries Research--1969* to NIDR's 1971 National Caries Program request for contracts, *Opportunities for Participation in the National Caries Program.* (Text from both documents is contiguous. Verbatim text is bolded.)**	
**ISRF 1969 Submission to NIDR** [1]	**NIDR 1971 National Caries Program RFC** [2]
concentrated preparations of dextranase have been made available and at present are undergoing clinical trial for their ability to reduce plaque formation in humans when applied topically.<br>A report in press shows that addition of a dextran of low molecular weight to a high sucrose diet significantly lowered the caries rate in hamsters, presumably because it blocked combining sites on the enzyme dextranase and prevented synthesis of dextran of high molecular weight. This observation offers another possibility for control of plaque formation and consequent development of caries. Finally an immunological approach is suggested by a recent report on neutralization of dextranase by homologous antibodies.	designated as Streptococcus mutans. In 5 percent sucrose broth this organism grows in coherent masses adhering to glass, teeth, or stainless steel wires. This property results from the activity of a dextransucrase, which by transglycosylation converts sucrose into its fructose moiety and an extracellular, water insoluble, adhesive, generally referred to as a dextran.<br>**Present evidence indicates that the greater cariogenicity of S. mutans, compared with a variety of other oral acidogens, relates to its characteristic of producing insoluble dextrans of high molecular weight** which accounts for the greater adhesiveness of **S. mutans to the tooth surface**.<br>Dextran provides as much as 10 percent of the dry weight of plaque, or a third of plaque matrix. In the oral cavity, dextran exists as a gel which when acidulated by metabolic end products of plaque bacteria, may help initiate natural caries.<br>**Incorporation of a dextranase preparation in the drinking water** of **hamsters reduced plaque** accumulation and **caries** even though the animals were **on a high sucrose diet** and harbored S. mutans. Human plaque, however, is only partially composed of dextran. Thus, the results of animal experiments cannot arbitrarily be assumed to apply in the human. It will be necessary to run controlled clinical trials for several years to determine the efficacy of dextranase or similar enzymes in reducing caries increment in humans.<br>Another aspect of this area of research is the effect of incorporating dextran of low molecular weight (15,000-20,000) in the diet. In hamsters subsisting on a high sucrose diet low molecular weight dextran was found to be effective in reducing plaque accumulation and caries. By providing an alternative glucosyl acceptor, the low molecular weight dextran presumably diverted the reaction of dextransucrase and sucrose away from synthesis of insoluble high molecular weight dextran. This too will require clinical testing before proper evaluation can be made.<br>We want to know:<br>1. Can mechanical cleansing agents and techniques be developed which will effectively disperse or prevent bacterial

**Table S1: Comparison of ISRF's submission to the NIDR Caries Task Force: *Dental Caries Research--1969* to NIDR's 1971 National Caries Program request for contracts, *Opportunities for Participation in the National Caries Program.* (Text from both documents is contiguous. Verbatim text is bolded.)**	
**ISRF 1969 Submission to NIDR** [1]	**NIDR 1971 National Caries Program RFC** [2]
	deposits on teeth?<br>2. Will plaque-dissolving agents such as dextranase or other enzymes reduce caries incidence in humans?<br>3. Will low molecular weight dextrans or similar products prevent the formation of sucrose-caused plaque?
For 20 **years** it has been known that certain antibiotics can **reduce the incidence of caries** in **rats**, hamsters, and humans. Yet dentistry has been strangely reluctant to exploit this promising lead—possibly because the microbial target was not well enough defined, possibly because of concern about possible deleterious changes in the oral microbiota, possibly because of unfavorable effects of certain antibiotics, such as tetracyclines, on the teeth. Now the target is more nearly defined, and bacteriological studies indicate that **long term administration of** penicillin, for example, **does not alter the oral flora harmfully**. Some dental researchers feel that a vigorous program to develop rational use of **antibiotics** or other antimicrobial agents **in oral hygiene** is overdue. Much evidence indicates that even partial or selective reduction of plaque-forming oral bacteria would go far to diminish caries.<br>A scientist at the National Institute of Dental Research suggests **the following criteria** to guide the selection of **antimicrobial agents** for topical **application** to prevent caries. The agents should be **effective against** homolactic **streptococci and lactobacilli,** and therefore as a rule would inhibit a variety of other gram-positive bacteria. Drug **resistant mutants** should occur rarely, if at all. It would be very desirable to select agents unlikely to come into general use orally or parenterally for **systemic** disease. Accordingly, they should **not** be **absorbable through the oral mucosa or** from the **gastro-intestinal tract**. Preferably they should be destroyed in the stomach or intestine, to reduce the chance of altering the intestinal flora. They should be palatable, **harmless to** oral **mucosa and teeth**, and nonallergenic. They should have a long shelf life, particularly in solution. The NIDR scientist feels that observance of these criteria should overcome the sort of opposition that has met proposals for intraoral use of some of the more popular antibiotics.	<u>Inhibiting Growth</u><br>Nearly 25 **years** ago, the principle was established that administration of a chemotherapeutic agent (penicillin) to **rats** via the food and drinking water could greatly **reduce the incidence of caries** and, incidentally, the oral count of lactobacilli. Similar findings were made in children receiving 200,000 units of penicillin by mouth daily for rheumatic fever prophylaxis. Over an average period of 4-5 years, during which their permanent teeth erupted, the children on the antibiotic had significantly less caries than public school children not on antibiotics. It has been observed that **long term administration of** antibiotics **does not** necessarily **alter the oral flora harmfully**, thus pointing to the possible use of **antibiotics in oral hygiene.**<br>The potentialities of antibacterial chemicals ("antiseptics") also needs exploration. In many respects these agents may be the best of the antimicrobials. As they do not have a specific spectrum the antiseptic agent could be expected to hold the oral biota in check overall with less risk of altering its normal balance deleteriously.<br>It may also be feasible to control caries-conducive activities of plaque bacteria without resorting to a direct attack on their viability. Theoretically one could find metabolic regulators (antimetabolites) that would alter, for example, bacterial utilization of cariogenic substrates such as sugars, the production of acids, the formation of adherent extracellular polysaccharides, or the accumulation of intracellular polysaccharides.<br>The use of an antimicrobial agent in the prevention of caries does not necessarily include a requirement for frequent application. Rats receiving a cariogenic diet containing 0.05 percent penicillin only 1, 2, or 3 days a week developed significantly less caries than untreated rats, though continuous administration of the antibiotic diet afforded much greater protection. In hamsters, after seven successive daily topical applications of 10 percent aqueous vancomycin to

Table S1: Comparison of ISRF's submission to the NIDR Caries Task Force: *Dental Caries Research--1969* to NIDR's 1971 National Caries Program request for contracts, *Opportunities for Participation in the National Caries Program.* (Text from both documents is contiguous. Verbatim text is bolded.)	
**ISRF 1969 Submission to NIDR** [1]	**NIDR 1971 National Caries Program RFC** [2]
	the teeth S. mutans could no longer be recovered by direct culture during the remaining 44 days of the experiment; plaque formation and caries were negligible.<br>Such results suggest that it might be possible to control the human oral flora adequately by infrequent but regular intraoral **application** of suitable **antimicrobial agents**. However, it is necessary to avoid indiscriminate dosing. Antimicrobial agents for topical application to prevent caries must be carefully selected. **The following criteria** have been suggested as defining an ideal agent for this purpose: 1. not likely to be administered for control of **systemic** diseases (preferably **not absorbable through the oral mucosa or gastrointestinal tract**); 2. free from systemic toxicity, non-allergenic, and **harmless to teeth and mucosa**; 3. **effective against** cariogenic **streptococci and lactobacilli** without the development of **resistant mutants**; 4. will not allow overgrowth of gram negative oral bacteria and yeasts; 5. will not be deactivated by saliva or by oral materials; 6. stable under necessary conditions of use and organoleptically acceptable; 7. biodegradable in the environment of waste disposal systems. A few tests in humans with agents meeting many of these criteria have been reported but there is need for considerable effort in this field.<br>The following information is needed:<br>1. Based on the criteria listed above, what antimicrobial agents (antibiotics, antiseptics, antimetabolites) are available for intraoral application in humans?<br>2. What is the most effective way (I.e., mouth rinses, gels, toothpaste, etc.) to use antimicrobial agents?<br>3. How effective are antimicrobial agent in reducing caries incidence in humans?
Recently, reports from two countries within weeks of one another indicated that research is actually a big step closer to preventing caries through **immunization**. One scientist developed a vaccine that is effective in rats, the other a vaccine effective in monkeys. An American cariologist reported that rats subjected to a new immunization procedure demonstrated 60% greater protection from caries than rats which had not been immunized. Protection was achieved by blocking	Immunization<br>It has been suggested that the cariogenic flora might be kept under control by active **immunization**, either against antigens of the bacterial cells proper or against antigenic bacterial products such as dextransucrase. Opposed to this concept is the fact that, unlike most infectious diseases, an attack of dental caries confers no resistance to a subsequent attack. It cannot be disputed, however, that about one person in a thousand remains free of caries

Table S1: Comparison of ISRF's submission to the NIDR Caries Task Force: *Dental Caries Research--1969* to NIDR's 1971 National Caries Program request for contracts, *Opportunities for Participation in the National Caries Program.* (Text from both documents is contiguous. Verbatim text is bolded.)

**ISRF 1969 Submission to NIDR** [1]	**NIDR 1971 National Caries Program RFC** [2]
production of dextranase. When the enzyme is injected into rats it is received as foreign matter and antibodies are formed against it. These antibodies remain in the body and block further production of the enzyme. At the same time a British dental scientist reported the development of a similar successful vaccine in monkeys. Both investigators noted that, although work is preliminary, prospects for preventing human tooth decay through immunization are encouraging.<br>It is thought that perhaps no one measure will suffice to control caries, but some combination of available and imminent measures may very likely do so.	indefinitely, seemingly despite exposure to cariogenic bacteria and diets. Such persons have often been designated as caries-immune. The basis for this natural freedom from caries has not yet been established, though it has been observed that it occurs more frequently among relatives as in the general population, and caries-free male adults oμtnumber females. Environmental fluoride apparently is not a factor as caries-free adults also are found in low-fluoride regions.<br>The leukocytes from caries-resistant subjects were found, in many instances, to phagocytize cariogenic streptococci to a significantly greater extent than did the leukocytes from caries-active individuals, although a specific antibody has not been found. Abundant evidence has been accumulated showing that various antibacterial antibodies occur in whole saliva, though their origin and immunoglobulin class have been identified in few cases. If it can be proven that the salivary system responds to local antigenic stimulus and secretes homologous antibody into the oral cavity where it combines with oral bacteria, then the case for immunization against dental caries is strengthened. Such antibody, for example, might hinder plaque accumulation by altering the surfaces of bacteria so that they would not adhere to the teeth.<br>Answers to a number of questions in this area are needed:<br>1. Can the bacteria cariogenic to humans be identified and what are their serological groupings?<br>2. Can local antibody formation in regional lymph nodes, other lymphoid tissues, and salivary glands be established as a consequence of local administration of antigens from cariogenic bacteria?

REFERENCES

1. International Sugar Research Foundation (1969) Dental caries research—1969. In: Roger Adams: an inventory of the papers of Roger Adams at the University of Illinois Archives. Record Series Number 15/5/23. Urbana (Illinois): University of Illinois Archives.
2. National Institute of Dental Research (1971) Opportunities for Participation in the National Caries Program. Available: http://catalog.hathitrust.org/Record/003436264. Accessed 16 May 2014.
